# Can We Really Believe This Platelet Count?

**DOI:** 10.1002/ajh.70172

**Published:** 2025-12-27

**Authors:** James Manning, Simona Deplano, Ketan Patel, Barbara J. Bain

**Affiliations:** ^1^ Department of Haematology Imperial College Healthcare NHS Trust, Hammersmith Hospital London UK; ^2^ Faculty of Medicine, Centre for Haematology St Mary's Hospital Campus of Imperial College London London UK


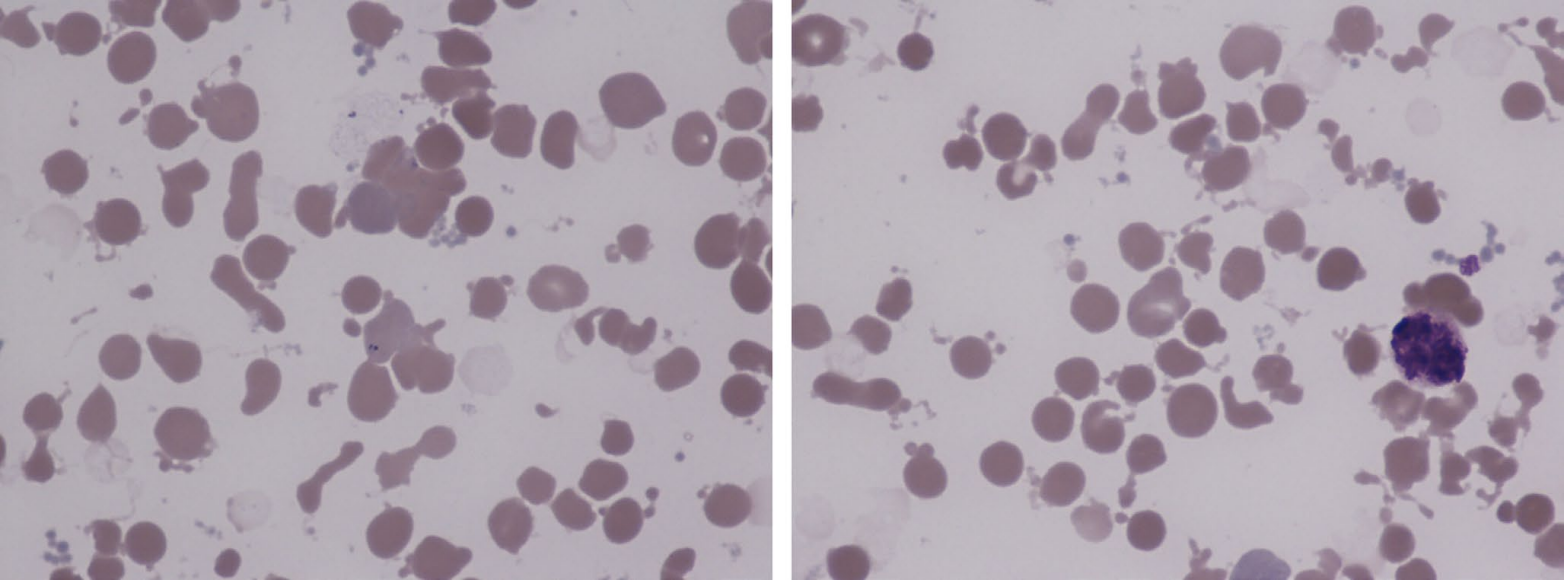


A 64‐year‐old woman presented to the hematology clinic for follow up of refractory chronic cold agglutinin disease with hepatic iron overload secondary to a chronic red cell transfusion burden. Management had included single‐agent rituximab and ciclosporin, to which the disease had proven refractory. She had recently received a first cycle of bendamustine and rituximab. On review preceding a second cycle, she was tired and lightheaded and scleral icterus was noted. A blood sample was taken urgently and conveyed to the laboratory for testing. Full blood count results, compared to results from three weeks earlier, are tabulated.

**Table:** 

	Current	Previous
White blood cell count (WBC) × 10^9^/L	35.6	4.2
Hemoglobin concentration (Hb) g/L	65	87
Mean cell volume (MCV) fL	80.6	95.2
Red cell distribution width (RDW) % [NR 10.0–15.9]	30.7	16.4
Platelet count × 10^9^/L	1125	444

Given the unexpected abnormalities in the blood count, an urgent blood film was made. This showed only small red cell agglutinates (left image, ×100 objective) and did not confirm the apparent thrombocytosis. The most striking feature was the presence of numerous red cell fragments, many of which were spherical and some of which were budding from red cells (both images). There were small numbers of more angular fragments. The thrombocytosis was factitious and attributable to schistocytes being counted as platelets. The leucocytosis was also factitious and attributable to loose clumps of platelets and debris being counted as leucocytes. All neutrophils were cytologically abnormal with blurred nuclear and cytoplasmic outlines and increased granularity (right image). The findings were not suggestive of a microangiopathic hemolytic anemia since the schistocytes were not often angular but rather were often microspherocytes and could be seen budding from red cells. These findings suggest the effect of heat. These distinctive heat effects can be seen both in patients suffering from burns and also when a blood sample has been inappropriately heated in vitro. Further investigation revealed that the phlebotomist had transported the patient's blood specimen to the laboratory in a mug of hot water.

Erroneous results of a blood count can result from preanalytical, analytical, or post‐analytical errors. This is an example of an uncommon preanalytical error. It is important that when it is necessary to keep a blood specimen warm it is kept at a controlled temperature of 37°C. Staff training is also important.

## Conflicts of Interest

The authors declare no conflicts of interest.

## Data Availability

Research data are not shared.

